# Epiregulin levels and their association with prognostic factors in Hodgkin lymphoma: a case-control study

**DOI:** 10.1007/s00277-026-06879-4

**Published:** 2026-02-10

**Authors:** Özden Yildirim Akan, İsmail Demir, Ferda Bilgir, Giray Bozkaya, Şerafettin Ceylan, Oktay Bilgir

**Affiliations:** 1 Health Sciences University, İzmir Faculty of Medicine, Bozyaka Training and Research Hospital, Department of Internal Medicine, İzmir, Turkey; 2 Health Sciences University,İzmir Faculty of Medicine, Bozyaka Training and Research Hospital, Department of Internal Medicine, İzmir, Turkey; 3 İzmir Katip Çelebi Unıversıty Atatürk Educatıon and Research Hospital Department of Allergy Immunology, İzmir, Turkey; 4 Health Sciences University, İzmir Faculty of Medicine, İzmir City Hospital, Department of Biochemistry, İzmir, Turkey; 5 Department of Family Medicine, İzmir Alsancak Nevvar Salih IsgorenState Hospital, İzmir, Turkey; 6 Health Sciences University, İzmir Faculty of Medicine, İzmir City Hospital, Department of Hematology, İzmir, Turkey

**Keywords:** Hodgkin lymphoma, Epiregulin

## Abstract

Epiregulin, a polypeptide involved in inflammation, cell proliferation, tumor development, and tissue remodeling pathophysiology, plays a crucial role in the etiopathogenesis of malignancies. This prospective case-control study aimed to investigate the association between epiregulin levels and various clinical and laboratory parameters in HL patients to better understand its role in the disease. This study compared newly diagnosed 56 HL patients with 56 healthy controls to assess epiregulin levels in relation to factors such as cell type, staging, extranodal involvement, β2-microglobulin, lactate dehydrogenase (LDH), and C-reactive protein (CRP). Epiregulin levels were significantly elevated in HL patients (265.0 ± 36.0 pg/ml) compared to healthy controls (130.7 ± 21.8 pg/ml). While no significant differences were observed in epiregulin levels among different HL subtypes, higher levels were found in advanced stages (281.1 ± 32.2 pg/ml) compared to early stages (246.4 ± 31.3 pg/ml). Patients with splenic and extranodal involvement exhibited elevated epiregulin levels. Furthermore, a positive correlation was noted between epiregulin levels and CRP, LDH, and β2-microglobulin levels in HL patients. Epiregulin levels are elevated in patients with Hodgkin lymphoma and are associated with advanced disease stage, extranodal and splenic involvement, the presence of B symptoms, and key laboratory parameters. These findings suggest that epiregulin may be related to disease burden and activity in HL. Further prospective studies are warranted to clarify its clinical relevance.

**Supplementary Information:**

The online version contains supplementary material available at 10.1007/s00277-026-06879-4.

## Introduction

Epiregulin, a 46-amino acid polypeptide growth factor, interacts with the epidermal growth factor receptor (EGFR) and v-erb-b2 oncogene homolog (ERBB) receptors, thereby influencing processes such as inflammation, cell proliferation, angiogenesis, and tissue remodeling [1]. Its association with various cancers, including those of the head and neck, bladder, stomach, colon, breast, lung, and liver, implicates it in tumor progression and metastasis [2, 3]. This suggests that epiregulin may warrant further investigation in the context of hematologic malignancies.

Hodgkin lymphoma (HL) is a lymphoid neoplasm characterized by the presence of Reed-Sternberg cells. It constitutes approximately 10% of all lymphoma cases in the United States. In 2018, an estimated 8,500 new cases of HL were diagnosed, with 1,050 resulting in death. The incidence is higher among White individuals, peaking at ages 20–24 and 75–79, and predominant in males [4]. In 2020, HL had an incidence rate of 0.98 and a mortality rate of 0.26 per 100,000. Despite a decrease in mortality, HL incidence has been on the rise, particularly among females, younger populations, and individuals from Asian countries [5].

Around 90% of HL cases are classified as classical HL, with subtypes like Nodular sclerosis, Mixed cellularity, Lymphocyte-rich, and Lymphocyte-depleted, while the remaining 10% are Nodular Lymphocyte-Predominant B-cell Lymphoma [6]. Etiological factors for HL include Epstein-Barr Virus, environmental influences, socioeconomic status, immunodeficiencies, autoimmune diseases, and genetic factors^4^. The tumor microenvironment, including immune interactions, is crucial for tumor growth and immune evasion. Research suggests that altered enzymatic pathways, cytokines, growth factors, and inflammatory molecules contribute to HL development [7].

Epiregulin, a polypeptide with established roles in inflammation, cell proliferation, and angiogenesis, has been identified as a significant factor in the development and progression of epithelial-origin malignancies. While several studies have explored its functions in these contexts, the investigation of Epiregulin in hematologic malignancies, such as HL, as well as its relationship with cell types in HL, clinical stages, and other laboratory parameters remains unexplored.

## Methods

### Ethical approval and study design

The case-control study was approved by the Ethics Committee of Bozyaka Training and Research Hospital(21.12.2022 Decision number:176) and included 56 newly diagnosed HL patients aged 18–65 years and 56 healthy controls. All participants provided written informed consent, and the study followed the 2013 Helsinki Declaration.

The study was conducted at Bozyaka Training and Research Hospital between 2023 and 2024. Patients were stratified according toβ2-microglobulin levels (< 3.5 mg/L or ≥ 3.5 mg/L, based on ROC curve analysis), disease stage, and extranodal involvement.

Exclusion criteria included diabetes, heart disease, thyroid disorders, COPD, pregnancy, liver/kidney disease, other cancers, and autoimmune diseases with chronic medication use.

### Biochemical analyses

Volunteers provided venous blood samples after a 10-hour fast for hemogram, sedimentation, CRP, LDH, beta-2 microglobulin, and biochemical tests. Initial analyses were performedon the same day. For Epiregulin measurement, blood was centrifuged at 2000 g for 10 min, and serum was stored at -80 °C. Serum Epiregulin levels were measured using an ELISA kit (Cusabio, Inc.) with a sensitivity of 3.9 pg/mL and detection range of 15.6 to 1000 pg/mL, maintaining a precision CV% below 10%.

### Statistical analysis

Power analysis determined a study population of 56 subjects per group. Data was analyzed using SPSS software, with Kolmogorov-Smirnov test for variable distribution, Chi-square tests for categorical variables, and Student’s t-test for demographic and laboratory characteristics. One-way ANOVA compared epiregulin levels across HL subtypes, and ROC curve analysis assessed the predictive value of circulating epiregulin levels for HL. Results with a p-value < 0.05 were considered statistically significant.

## Results

The study included 112 volunteers, with 56 in the control group and 56 with HL. In the control group, 57.1% were male and 42.9% female, while in the HL group, 53.6% were male and 46.4% female, with no significant gender difference (χ2 = 0.284; *p* = 0.594). The mean age was 35.9 ± 14.7 years for controls and 37.6 ± 16.8 years for HL, with no significant difference *(p = 0.443).* Comparison of parameters among control and HLgroup is shown in Table 1.

**Table 1:** Comparison of parameters among control and HL

Variables	Control (*n* = 56)	HL (*n* = 56)	*p* value
	Mean ± SD		
Age (Years)	35.9 ± 14.7	37.6 ± 16.8	0.447
WBC, x1000/µL	7.7 ± 1.5	7.8 ± 4.9	0.890*
HGB, g/dL	13.8 ± 2.0	11.7 ± 4.1**↓**	***0.002***
HCT, %	42.4 ± 5.0	39.5 ± 6.4	*0.007*
PLT, x1000/µL	286.9 ± 86.3	265.3 ± 96.1	0.213
RDW-CV, %	12.9 ± 1.7	14.8 ± 2.4**↑**	***< 0.001***
NEU, x1000/µL	4.3 ± 1.4	4.8 ± 4.4	0.415
LYM, x1000/µL	2.6 ± 0.8	4.0 ± 1.2**↑**	***< 0.001***
PDW, %	12.5 ± 2.1	11.6 ± 2.0**↓**	***0.017***
Glucose, mg/dL	95.9 ± 14.8	96.1 ± 9.4	*0.951*
Sedim, mm/h	15.4 ± 12.8	30.8 ± 23.3**↑**	< 0.001*
HbA1c, %	5.5 ± 0.7	5.3 ± 0.4	***0.048***
CRP, mg/L	2.9 ± 4.3	16.1 ± 19.5**↑**	< 0.001*
Urea, mg/dL	26.0 ± 7.2	30.1 ± 22.6	0.195*
Creatinin, mg/dL	0.80 ± 0.18	0.74 ± 0.25	0.207
AST, U/L	21.9 ± 17.0	28.3 ± 25.8	0.123*
ALT, U/L	24.9 ± 21.9	43.0 ± 62.3**↑**	0.044*
Albumin, g/L	4.7 ± 3.5	4.5 ± 5.0**↓**	0.008*
TSH, mU/L	2.1 ± 1.4	2.0 ± 1.4	0.602
Ferritin, µg/L	96.4 ± 108.2	337.1 ± 611.1**↑**	0.005*
Total Bilirubin, mg/dL	0.51 ± 0.27	0.60 ± 1.68	0.709*
LDH, U/L	219.5 ± 65.2	502.6 ± 176.5**↑**	***< 0.001***
Epiregulin (pg/mL)	130.7 ± 21.8	265.0 ± 36.0**↑**	***< 0.001***

Independent t test and *Mann-Whitney U test used. *p* < 0.05 considered significant. ↑↓ Symbols indicate whether higher or lower than the control group

Abbreviations: HL, Hodgkinlymphoma; WBC, White blood count; HGB, Hemoglobinblood test; HCT, Hematocrit blood test; PLT, Platelet blood test; RDW-CV, Red cell distribution width; NEU, Neutrophil blood test; LYM, Lymphocytesbloodtest; PDW, Platelet distribution width; HbA1c, Glycosylated hemoglobin; CRP, C-reactiveprotein, ALT, Alanine aminotransferase; AST, Aspartate aminotransferase; TSH, Thyroidstimulating hormone; LDH, Lactate dehydrogenase

**Fig. 1:**
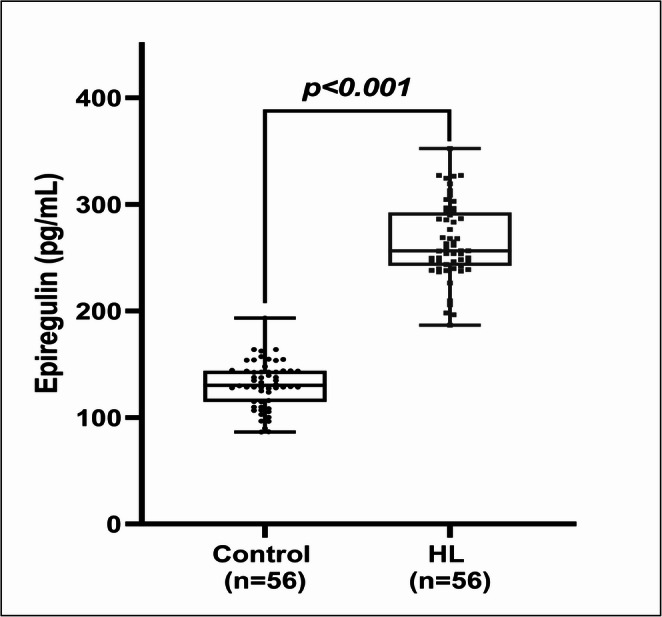
Comparison of epiregulin levels among control and HL

Epiregulin levels were significantly higher in the HL group compared to controls (*265.0 ± 36.0 vs. 130.7 ± 21.8 pg/mL, p < 0.001*) (Fig. 1).

Receiver operating characteristic (ROC) curve analysis was performed to determine the optimal epiregulin cut-off value within the Hodgkin lymphoma (HL) group. The area under the curve (AUC) was 1.000 (95% CI: 0.999–1.000, *p* < 0.0001). A cut-off value of 190.0 pg/mL was identified, yielding a sensitivity of 98.2% and a specificity of 98.2%. However, these findings should be interpreted with caution, as the ROC analysis was exploratory in nature and based on a limited sample without external validation. Further prospective, large-scale, and multicenter studies are required before epiregulin can be defined as a prognostic threshold.

**Fig. 2:**
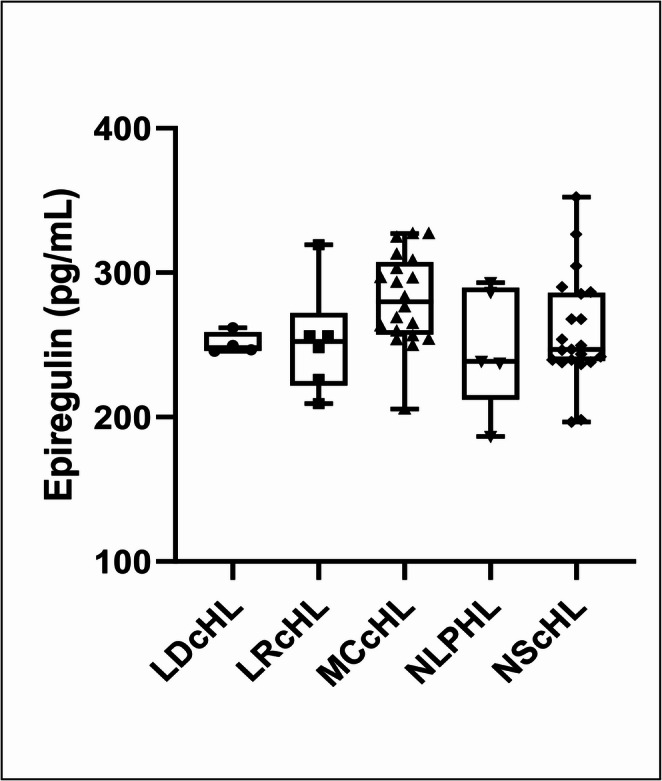
Comparison of epiregulin levels among HL cell subtype

The HL patient group included the following subtypes: LDcHL (*n* = 4), LRcHL (*n* = 6), MCcHL (*n* = 20), NLPHL (*n* = 5), and NScHL (*n* = 21). No significant difference in Epiregulin levels was found between the subtypes *(p = 0.136)* (Fig. 2).

There was no significant difference in epiregulin levels between males and females *(p = 0.211)*, though females had slightly higher levels. A significant difference was observed based on a β2 Microglobulin cut-off of 3.5 mg/L, with higher epiregulin levels in individuals with levels ≥ 3.5 mg/L *p = 0.010)* (Table 2). Using the Youden’s index to determine the optimal cut-off point from the ROC curve, Epiregulin demonstrated a sensitivity of 81.6% and a specificity of 55.6% at a cut-off value of 246.0 pg/mL. The ROC analysis evaluated the diagnostic performance of Epiregulin in predicting B2M levels ≥ 3.5. The area under the curve (AUC) was 0.72, SE = 0.08, *p* = 0.008, 95% CI [0.57, 0.87], indicating a statistically significant discriminatory power. The analysis was based on 38 positive and 18 negative cases. Epiregulin levels were higher in stage 3&4 of HL compared to stage 1&2 (281.1 ± 32.2 vs. 246.4 ± 31.3 pg/mL, *p* < 0.001). Additionally, patients with extranodal involvement had higher epiregulin levels compared to those without.*(272.5 ± 31.7 vs. 246.1 ± 40.1 pg/mL, p = 0.012).* An independent samples t-test revealed that epiregulin levels were significantly higher in patients with B symptoms (274.2 ± 35.8) compared to those without B symptoms (241.7 ± 25.0), t(54) = -3.32, *p* = 0.002.

**Table 2:** Comparison of Epiregulin levels in different variables (gender, 2 microglobulin, extranodal HL) for HL group

Variables	Subgroups	Epiregulin (pg/mL)	*p* value
Gender			
	Male (*n* = 30)	257.6 ± 39.0	0.211
	Female (*n* = 26)	270.0 ± 33.5	
β2M, mg/L			
	β2M < 3.5 (*n* = 24)	248.2 ± 33.7	***0.010***
	β2M > = 3.5 (*n* = 32)	272.9 ± 34.7	
Stage			
	Stage 1–2 (*n* = 26)	246.4 ± 31.3	***< 0.001***
	Stage 3–4 (*n* = 30)	281.1 ± 32.2	
Extranodal HLSpleen			
	No (*n* = 40)	246.1 ± 40.1	***0.012***
	Yes (*n* = 16)	272.5 ± 31.7	
B symptoms			
	Yes (*n* = 40)	(274.2 ± 35.8 )pg/mL	***0.002***
	No (*n* = 16)	(241.7 ± 25.0), pg/mL	

Mann-Whitney U testused and *p* < 0.05 considered significant. β2M; β2 Microglobulin, HL; Hodgkin’s Lymphoma

Compared to the control group, the HL group showed significantly higher CRP levels, with a weak to moderate positive correlation between epiregulin and CRP (Spearman’s rs = 0.364; *p* = 0.006). A moderate positive correlation was also found between epiregulin and LDH (Spearman’s rs = 0.513; *p* = 0.009). In the HL group, a strong positive correlation was observed between epiregulin and B2M (Spearman’s rs = 0.745; *p* = 0.001)

Association between B-symptoms and Stage: The association between B-symptoms and disease stage group was not statistically significant (χ²(1) = 2.33, *p* = 0.127). Descriptively, the prevalence of B-symptoms was higher in the advanced-stage group (24/30, 80.0%) compared to the early-stage group (16/26, 61.5%).(Table 3).

**Table 3:** Descriptive statistics and comparison of epiregulin levels by B-symptoms status in early (Stages 1&2) and advanced (Stages 3&4) Hodgkin lymphoma

Statistics Term	Stage 1&2		Stage 3&4	
	B-Symptoms		B-Symptoms	
	No (*n*=10)	Yes (*n*=16)	No (*n*=6)	Yes (*n*=24)
Minimum	186.6	196.5	226.1	239.5
25% Percentile	208.5	237.5	235.2	262.1
Median	246.0	250.3	248.9	291.6
75% Percentile	250.7	266.6	267.9	311.8
Maximum	286.3	324.5	267.9	352.4
Range	99.70	128.0	41.80	112.9
Mean	237.0	252.2	249.6	288.9
Std. Deviation	28.72	32.33	16.46	30.43
Std. Error of Mean	9.081	8.083	6.719	6.211

**Fig. 3:**
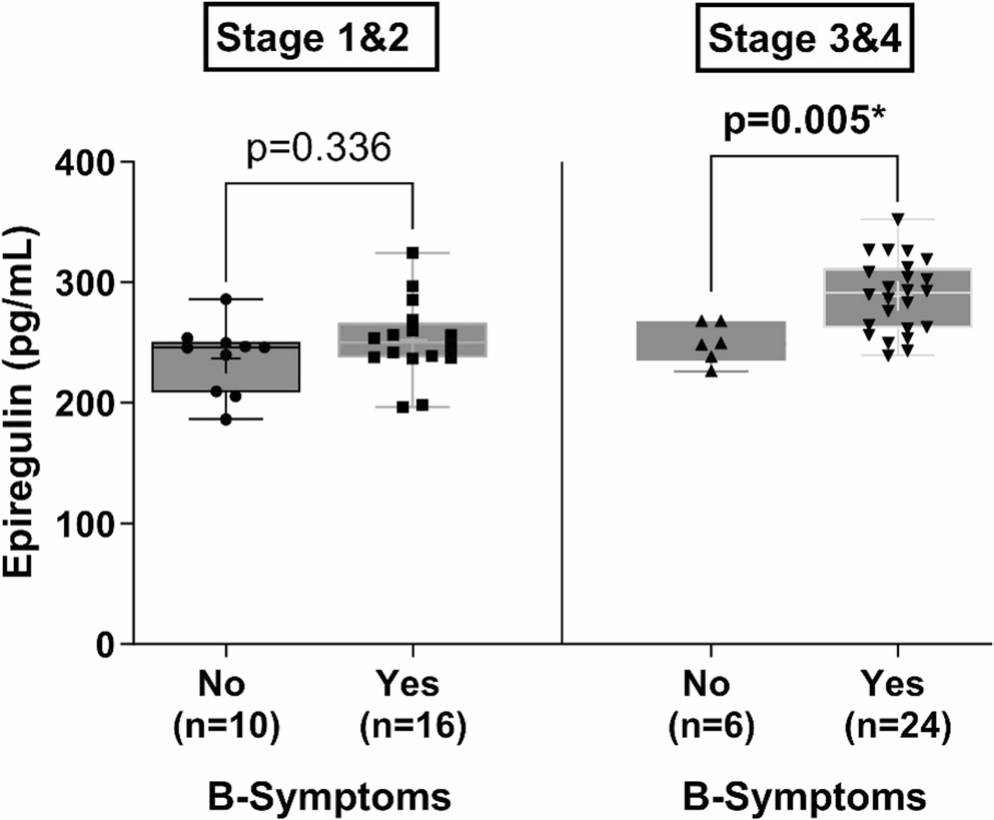
Comparison of epiregulin levels by B-symptoms status in early (Stages 1&2) and advanced (Stages 3&4) Hodgkin lymphoma. (Mann-Whitney U analysis used)

While Epiregulin levels were similar between patients with and without B-symptoms in Stage 1&2 (252.2 ± 32.3 pg/mL vs. 237.0 ± 28.7 pg/mL, *p* = 0.336), patients with B-symptoms in Stage 3&4 had significantly (*p* = 0.005) higher Epiregulin levels (288.9 ± 30.4 pg/mL vs. 249.6 ± 16.5 pg/mL) as shown in Fig. 3. This finding suggests that Epiregulin may be more strongly associated with the pathophysiology of B-symptoms in advanced-stage HL.

## Discussion

To our knowledge, this is the first study to explore the association between serum epiregulin levels and Hodgkin lymphoma in the context of a hematologic malignancy. The aim of the study was to compare epiregulin levels between newly diagnosed HL patients and healthy controls and to evaluate their relationship with lymphoma cell subtypes, disease stage, the presence of B symptoms, and selected laboratory parameters.

The primary finding of our study is significantly higher levels of serum epiregulin in HL patients compared to the controls. Although there were differences in epiregulin levels across HL cell types, no statistical significance was observed. Furthermore, epiregulin levels were significantly higher in the advanced stages of HL compared to the early stages. Additionally, patients with splenic and extranodal involvement, as well as those presenting with B symptoms, exhibited higher epiregulin levels compared to those without these clinical features.

Epiregulin, a member of the epidermal growth factor (EGF) family, acts as a ligand for EGFR and members of the ERBB (v-erb-b2 oncogene homolog) family of tyrosine kinase receptors, regulating cell growth and differentiation. EREG/EGFR factors impact cellular processes such as proliferation, invasion, metastasis, angiogenesis, and apoptosis resistance, contributing to aggressive tumor behavior^1^. Dysregulated EGF-ErbB signaling pathways are observed in human malignancies [8]. McIntyre et al. found increased Epiregulin expression in advanced-stage breast cancer patients with bone or lung metastases [9]. Additionally, Bapat et al. Suggested targeting Epiregulin, significantly upregulated in Tumor-associated macrophages in breast cancer, along with JAK inhibitors could limit macrophage-mediated therapeutic resistance, proposing Epiregulin as a potential therapeutic target polypeptide [10].

Recent studies have highlighted the role of epiregulin in lung cancer. Polycyclic aromatic hydrocarbons in cigarette smoke can elevate epiregulin expression, promoting cell proliferation and contributing to lung adenocarcinoma progression [11]. Additionally, Zhang and colleagues showed that neutralizing anti-epiregulin antibodies reduced lung cancer metastasis and invasion, improving prognosis and survival [12].

In colorectal cancer it has been shown that high levels of amphiregulin and epiregulinare associated with a positive response to anti-EGF therapies [13]. Wang and colleagues further proposed that targeting EGF within the tumor microenvironment, particularly by antagonizing epiregulin, could reduce its expression and consequently limit tumor proliferation and invasion. They also proposed that this approach could hold a potential for therapeutic application in various malignancies [14]. Additionally, studies have demonstrated that using EGFR antagonists in cancer treatment can decrease resistance to therapies and increase the effectiveness of treatment, making it a promising strategy for improving patient outcomes [15].

In hematological malignancies, the relationship between epiregulin and multiple myeloma has been studied. Liu and colleagues identified the role of epiregulin (EREG) in regulating the proliferation and metastasis of myeloma cells [16]. In a similar study, it was also noted that the EGF family ligands play a crucial role as growth factor activities for myeloma cells [17]. Research by Demir et al. revealed elevated epiregulin levels in non-Hodgkin lymphoma patients, highlighting the significance of EGF in hematologic malignities and the link between epiregulin and cancer progression [18].

The Epstein-Barr virus (EBV) infects over 90% of the global population and is associated with cancers such as Burkitt lymphoma, HL, and nasopharyngeal carcinoma (NPC). EBV maintains viral persistence by disrupting immune cell function, apoptotic pathways, and antigen processing. Additionally, EBV plays a role in the activation and transformationof B cells into immortalized cell forms that serves as a valuable model for viral latency and lymphoma development [19]. Studies have shown that the EBV oncoprotein LMP1 (Latent Membrane Protein 1) affects PKCδ, leading to the transcription of EGFR, which is highly expressed in nasopharyngeal carcinoma [20].

In our study, we observed a significant rise in epiregulin levels in the serum of newly diagnosed HL patients compared to healthy individuals. Our results indicated notably increased epiregulin levels in advanced-stage HL patients, particularly those with extranodal and splenic involvement, indicating escalating Epiregulin expression with disease progression. This aligns with existing research linking elevated epiregulin levels to advanced-stage cancers and metastases. These findings suggest that epiregulin may be associated with disease burden and progression in Hodgkin lymphoma and warrant further investigation.

Extensive research has documented the link between growth factors and processes such as inflammation, angiogenesis, and tissue remodeling. Conditions like inflammatory connective tissue diseases and multiple sclerosis have been associated with increased serum levels of various growth factors, including epiregulin, amphiregulin, TGF-α, fibroblast growth factor 2, tenascin C, interleukins, and CRP [21]. Epiregulin is pivotal in regulating inflammation, wound healing, angiogenesis, and vascular remodeling. Evidence indicates that molecules such as angiotensin II, endothelin-1, and antithrombin promote the cleavage of the transmembrane epiregulin proform, resulting in the release of the mature epiregulin form during inflammation [22]. In research by Negreiros et al., elevated CRP levels have been associated with advanced disease stages and the presence of B symptoms. Additionally, CRP levels have been recognized as a potential marker of treatment response, with persistently high CRP values linked to resistance to HL treatment [23]. Vassilakopoulos et al. also emphasized the importance of beta-2 microglobulin as a significant molecular marker, serving as an independent prognostic factor in HL [24].

Our analysis further identified a significant correlation between the presence of B symptoms and elevated serum epiregulin levels. While the overall prevalence of B symptoms did not differ markedly across various disease stages, patients with advanced-stage HL who exhibited B symptoms demonstrated significantly higher epiregulin concentrations compared to those without B symptoms. This observation implies that epiregulin may be implicated in mechanisms associated with systemic symptoms such as fever, weight loss, and night sweats, potentially due to its pro-inflammatory and angiogenic properties [21]. This novel finding indicates that increased epiregulin levels are associated not only with advanced stages and extranodal involvement but also with systemic disease symptoms. Collectively, these findings suggest that epiregulin may reflect overall disease activity and severity in HL.

## Conclusion

In patients with Hodgkin lymphoma (HL), epiregulin levels are found to be elevated. These levels are particularly higher in individuals with advanced HL stages and those with splenic and extranodal involvement. Additionally, patients who exhibit B symptoms have been shown to have greater serum epiregulin levels than those without these symptoms. The positive correlation observed between epiregulin and prognostic markers such as C-reactive protein (CRP), lactate dehydrogenase (LDH), and β-2 microglobulin suggests a possible link between epiregulin and the disease burden and activity in HL. To better understand the clinical implications of epiregulin in HL, further prospective multicenter studies are necessary.

## Limitations

This study provides valuable preliminary data on the relationship between epiregulin and Hodgkin lymphoma; however, certain limitations should be acknowledged. The sample size, though statistically adequate, remains relatively limited for subgroup analyses. The study design was cross-sectional, and epiregulin levels were evaluated only at diagnosis, preventing assessment of changes during treatment or follow-up. Therefore, causal relationships between epiregulin levels and disease characteristics cannot be established.

The main drawbacks are the lack of follow-up measurements to demonstrate the kinetics of epiregulin level changes in relation to treatment response, and the absence of data regarding whether serum epiregulin concentrations correlated with therapeutic outcomes. Additionally, as this was a single-center study, future multicenter and longitudinal studies with larger patient populations are encouraged to validate and extend these findings.

## Supplementary Information

Below is the link to the electronic supplementary material.

Supplementary Material 1 (DOCX 27.0 KB)

## Data Availability

The data and materials can be found from the corresponding author.
