# Supplementary figures and images for: Epiregulin levels and their association with prognostic factors in Hodgkin lymphoma: a case-control study
Source: Ann Hematol. 2026 Feb 10;105(3):104. doi: 10.1007/s00277-026-06879-4 (PMC12891035; doi:10.1007/s00277-026-06879-4)

Figure Determination of Epiregulin cut-off value in the HL group; ROC analysis.
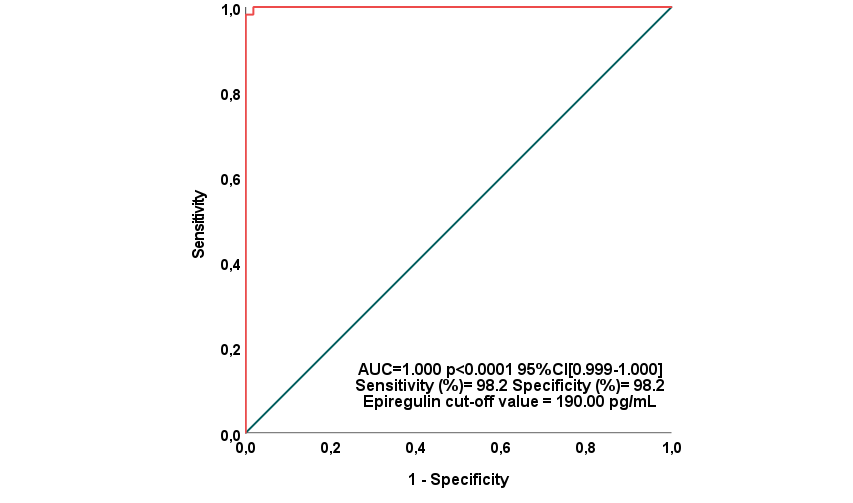

Supplement: Supplementary file 1 — Supplementary Material 1 (DOCX 27.0 KB) [file 277_2026_6879_MOESM1_ESM.docx]
